# A novel method for studying the temporal relationship between type 2 diabetes mellitus and cancer using the electronic medical record

**DOI:** 10.1186/1472-6947-14-38

**Published:** 2014-05-09

**Authors:** Adedayo A Onitilo, Rachel V Stankowski, Richard L Berg, Jessica M Engel, Gail M Williams, Suhail A Doi

**Affiliations:** 1 Department of Hematology/Oncology, Marshfield Clinic Weston Center, 3501 Cranberry Boulevard, Weston, WI 54476, USA; 2 Marshfield Clinic Research Foundation, Marshfield, WI, USA; 3 School of Population Health, University of Queensland, Brisbane, Australia; 4 Department of Hematology/Oncology, Marshfield Clinic Cancer Care at St. Michael’s Hospital, Stevens Point, WI, USA

**Keywords:** Type 2 diabetes mellitus, Cancer, Pre-diabetes, Electronic medical record, Method

## Abstract

**Background:**

We developed an algorithm for the identification of patients with type 2 diabetes and ascertainment of the date of diabetes onset for examination of the temporal relationship between diabetes and cancer using data in the electronic medical record (EMR).

**Methods:**

The Marshfield Clinic EMR was searched for patients who developed type 2 diabetes between January 1, 1995 and December 31, 2009 using a combination of diagnostic codes and laboratory data. Subjects without diabetes were also identified and matched to subjects with diabetes by age, gender, smoking history, residence, and date of diabetes onset/reference date.

**Results:**

The final cohort consisted of 11,236 subjects with and 54,365 subjects without diabetes. Stringent requirements for laboratory values resulted in a decrease in the number of potential subjects by nearly 70%. Mean observation time in the EMR was similar for both groups with 13—14 years before and 5–7 years after the reference date. The two cohorts were largely similar except that BMI and frequency of healthcare encounters were greater in subjects with diabetes.

**Conclusion:**

The cohort described here will be useful for the examination of the temporal relationship between diabetes and cancer and is unique in that it allows for determination of the date of diabetes onset with reasonable accuracy.

## Background

The National Cancer Institute estimates that approximately 13.7 million Americans with a history of cancer were alive on January 1, 2012 [1] with over 1.5 million additional cases diagnosed each year [2]. Diabetes mellitus is even more prevalent, affecting 25.8 million people, or 8.3% of the population, in the United States [3]. Accordingly, it is not uncommon for the same individual to be diagnosed with the both conditions, potentially compounding both illnesses [4,5]. Diagnosis of cancer may make management of diabetes more difficult or conversely, diabetes may be predictive of poorer cancer outcomes [4,6-9]. Understanding the relationship between cancer and diabetes and the impact that one disease may have on the other may provide important insight regarding both health and survival and has become a key research priority.

Recent studies have shed considerable light on the potential physiological and clinical relationship between diabetes and cancer [10-12]. Diabetes and cancer share several important risk factors and attempting to define the relationship between the two diseases is additionally confounded by demographic and lifestyle characteristics as well as exposure to diabetes medications [13]. Cancer tends to be somewhat easier to study using information available in the electronic medical record (EMR) and various cancer registries. Studies of diabetes generally prove to be more difficult. Diabetes develops gradually and is characterized by progressive insulin resistance and hyperinsulinemia during the pre-diabetes phase followed by increasing hyperglycemia after clinical onset. Lifestyle modifications, medication use, and other treatment options are not usually initiated until after clinical diagnosis and many patients with diabetes go unrecognized for long periods of time. Reliance on administrative data, which indicates only when diabetes was recognized and diagnosed, not necessarily when it began, has precluded careful temporal analyses of the relationship between diabetes and cancer. Even so, EMRs have served as an important data source in initial studies of the relationship between cancer and diabetes. Limitations of such studies include imprecision in capture of diabetes onset date, inaccuracies in electronic data, difficulty in distinguishing between type 1 and type 2 diabetes, and biases inherent to retrospective and observational studies. Due in part to these limitations, the temporal and causal relationship between diabetes and cancer, if any, remains difficult to explore.

Numerous individual studies and meta-analyses have yielded important information regarding cancer risk following diabetes onset [10]. However, recent evidence suggests that the hyperglycemia characteristic of overt diabetes may be less important in promoting cancer risk than the hyperinsulinemia characteristic of the pre-diabetes phases [13,14]. Due to the powerful effects of insulin as a growth factor and the potential for hyperinsulinemia to impact cancer development, a number of studies have attempted to correlate insulin levels with cancer risk, finding some effect for certain cancer types [15-18]. However, little attention has been paid to the pre-diabetes phase specifically in patients known to progress to diabetes, and the long-term temporal relationship between the two diseases remains unclear. The purpose of this paper is to describe a unique method for determining date of onset of type 2 diabetes, even when onset of disease occurs prior to clinical recognition. This algorithm leverages the EMR to draw upon clinical, administrative, and laboratory data to accurately pinpoint the date of diabetes onset, exclude potential subjects with type 1 diabetes, and examine additional confounding factors, such as glycated hemoglobin (HbA1c) levels and medication exposure. Our study algorithm and methods are described in detail and compared to those published by other authors. Limitations and potential biases are also discussed.

## Methods

Marshfield Clinic is a multi-specialty, regional healthcare system in Wisconsin, USA. The Marshfield Clinic EMR contains data dating back to the 1960s and provides comprehensive information regarding all encounters with the Marshfield Clinic and cooperating hospitals, including St. Joseph’s Hospital in Marshfield, WI. In 2007, Wilke et al. [19] published an electronic algorithm for identifying patients with diabetes mellitus in the EMR. However, this algorithm was focused on a specific subset of Marshfield Clinic patients enrolled in the Personalized Medicine Research Project (PMRP) and could not accurately pinpoint date of clinical diabetes onset. In the present study, we took this algorithm a step further and developed matched cohorts of patients with and without type 2 diabetes who received care at the Marshfield Clinic to retrospectively examine the temporal relationship between diabetes and three different types of cancer, including breast, prostate, and colon cancer, as well as medication exposure and glycemic control. The study was approved by the Marshfield Clinic Scientific Review Committee and the Institutional Review Board and a waiver of subject consent was granted [study ID—ONI10711/78037].

### Subject selection

Patients diagnosed with type 2 diabetes between January 1, 1995 and December 31, 2009 were eligible for inclusion in the study. All potential subjects were required to be 30 years of age or older by the end of the study period and could not have any diabetes-related diagnoses or medication use prior to the study period. The pool of potential subjects was then divided based on whether or not they had any diabetes-related diagnostic codes during the study period. Patients with one or more diabetes-related codes during the study period comprised the pool of potential subjects for the cohort with diabetes. Patients with no diabetes-related diagnoses prior to the end of the study period comprised the pool of potential subjects for the cohort without diabetes.

**Figure 1:**
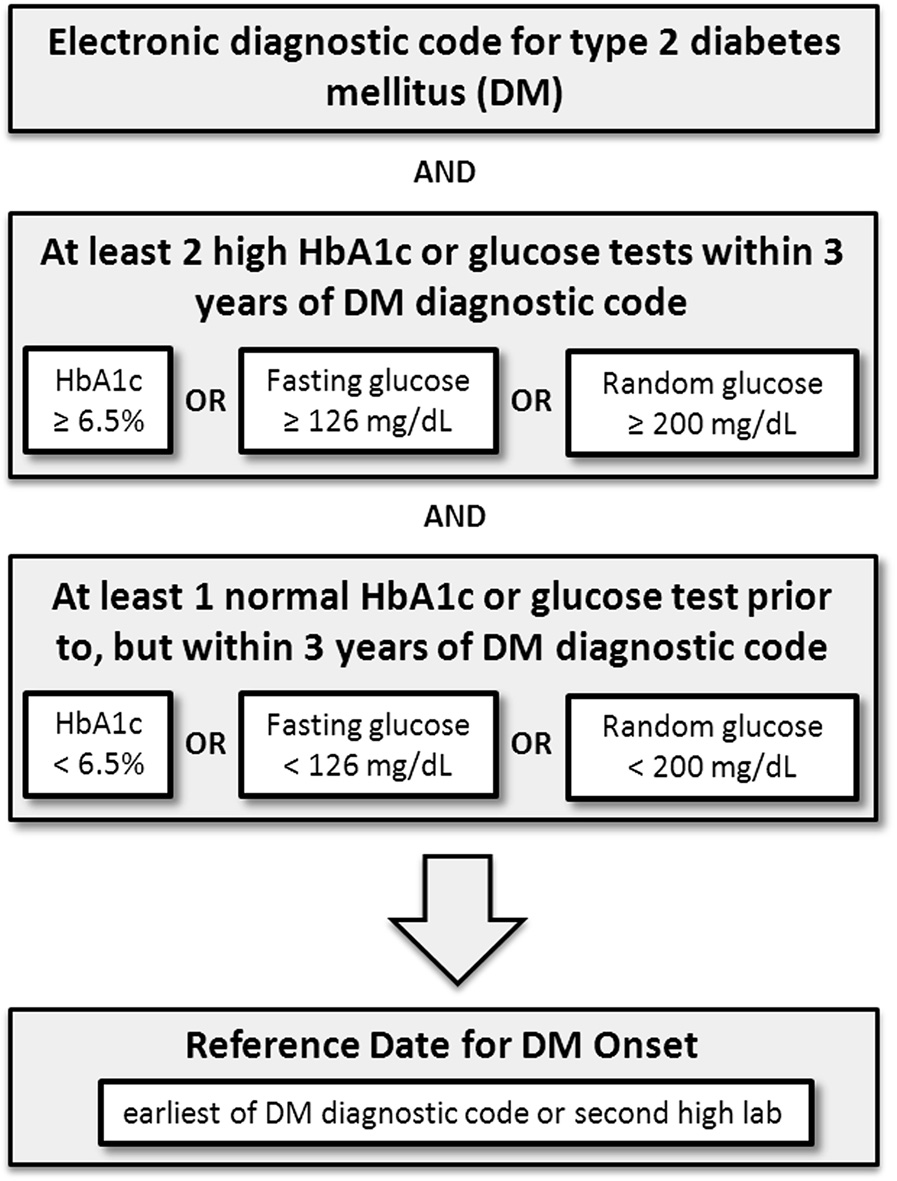
**Algorithm for defining type 2 diabetes.** Type 2 diabetes was defined using a combination of diagnostic and laboratory data. Laboratory results indicative of diabetes were based on American Diabetes Association criteria [20].

For study purposes, type 2 diabetes was defined using a combination of diagnostic codes and laboratory results (Figure 1). Data were collected electronically from the Marshfield Clinic EMR and cancer registry. Data validation was performed in an iterative fashion and included both electronic screening (e.g., graphical view of laboratory results, identification of seeming discrepancies between laboratory values and diagnoses) and manual review. Subjects manually reviewed included two sequential random samples (90 and 84 cases, respectively), with the selection stratified electronically with respect to prevalence of diabetes and cancer, calendar year, and location of residence. These samples were manually validated by search of the patient medical record for the presence and incident dates of diagnosis for diabetes and cancer through utilization of text records, diagnosis description codes, pathology reports, and review of diabetes-related laboratory values and medications. Validation results were used in refining the cohort definitions. Subjects with diabetes had at least one diagnostic code for type 2 diabetes mellitus (International Classification of Disease, version 9 (ICD-9) 250.X0 or 250.X2), and this code was required to precede any code for type 1 diabetes by at least one year. In addition, subjects with diabetes were required to have at least two abnormal laboratory test results for glycated hemoglobin (HbA1c) or glucose [HbA1c ≥ 6.5% (48 mmol/mol), fasting glucose ≥ 126 mg/ml, or random glucose ≥ 200 mg/ml] with the second being no more than three years prior to first type 2 diabetes diagnostic code, and at least one normal HbA1c or glucose test prior to, but within three years of first diabetes diagnostic code [HbA1c < 6.5% (48 mmol/mol), fasting glucose < 126 mg/ml, or random glucose < 200 mg/ml]. Laboratory criteria for type 2 diabetes were based on American Diabetes Association (ADA) criteria [20]. The date of diabetes onset was defined as the earlier of the first type 2 diabetes diagnosis by diagnostic code or the second high diabetes-related laboratory value. Requiring that both a normal result and the beginning of abnormal tests occurred within three years helps ensure capture of diabetes onset within that three-year window and excludes subjects with long-term, undiagnosed diabetes. Additionally, subjects treated with diabetes medications > 30 days before diagnoses were excluded. For the cohort with diabetes, the date of diabetes onset was considered the reference date. Subjects without diabetes were also verified by laboratory values and clinical data. Potential subjects without diabetes with no normal glucose or HbA1c test, as defined by the ADA, and those treated with diabetes medications prior to the end of the study period were excluded.

### Subject matching

**Figure 2:**
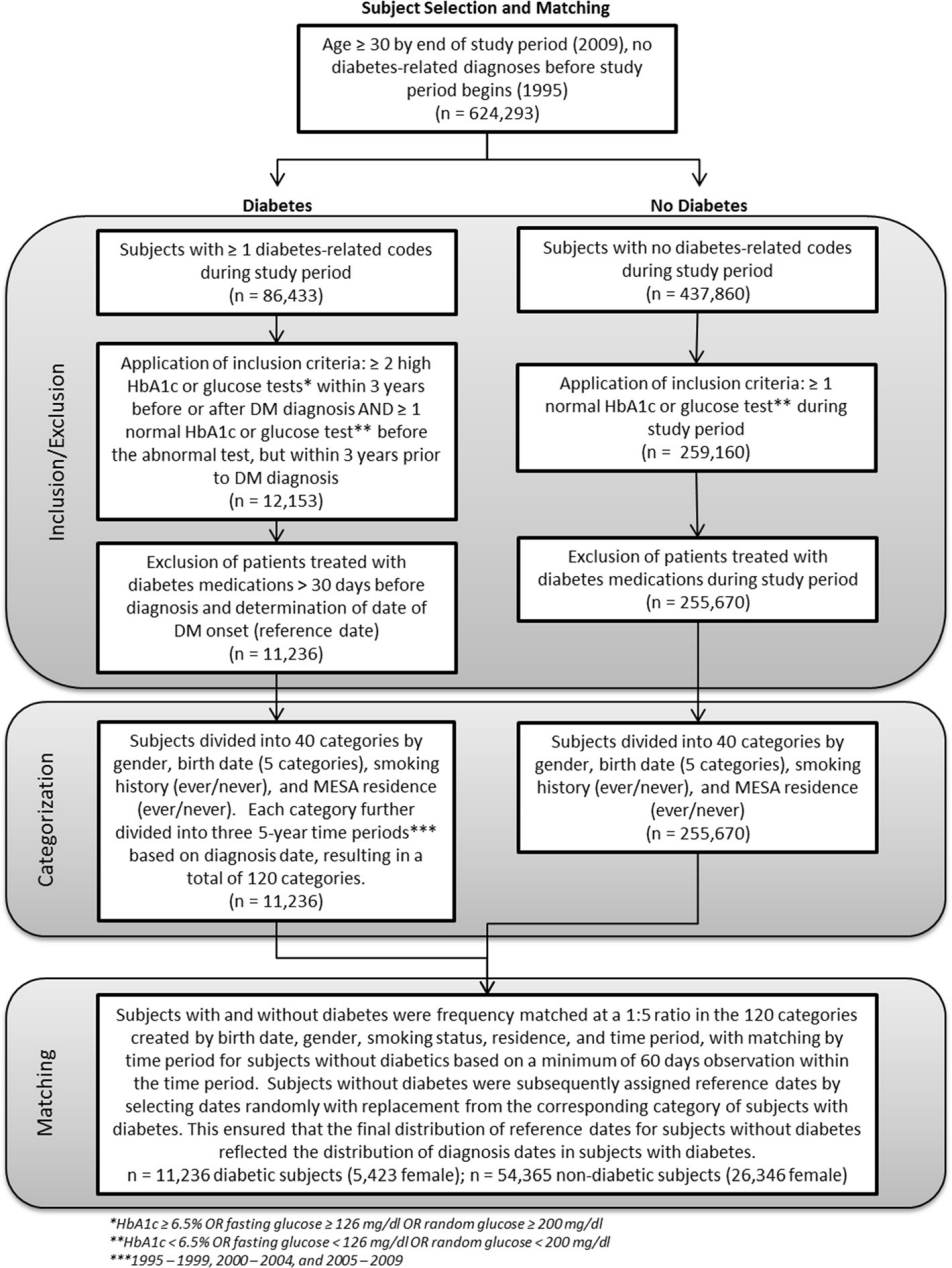
**Subject selection and matching.** Subject selection and matching process for defining cohorts of patients with and without type 2 diabetes.

Potential subjects without diabetes were frequency matched at a 5:1 ratio with subjects with diabetes based on date of birth (five categories), smoking history (ever/never), residence (ever/never) in the Marshfield Epidemiologic Study Area (MESA, a geographic region consisting of 14 ZIP codes in the primary service area of Marshfield Clinic [21]), and diabetes diagnosis/reference period (1995 – 1999, 2000 – 2004, or 2005 – 2009). Matching variables were selected based on the potential for effects on cancer risk and healthcare seeking behaviors. Additional baseline characteristics were accounted for via statistical adjustment. Initial subject selection required a minimum of 60 days observation time in the study period meaning that subjects had to have visits within the Marshfield Clinic system spanning at least the 60 days after the reference date. An actual reference date was assigned for each subject without diabetes by randomly sampling from the subset of subjects with diabetes in the same stratum, thereby ensuring that the distribution of possible observation times for subjects without diabetes corresponded precisely with those of their matched subjects with diabetes (Figure 2).

### Data collection

Data sources included Marshfield Clinic’s comprehensive EMR system and cancer registry [19]. Data were collected electronically and verified through manual chart abstraction of targeted samples. Reference dates for all subjects fell within the 15 year study period from 1995 through 2009, with follow-up through 2011 and observation before the reference date as far back as the patient’s history in the Marshfield Clinic EMR. Based on the need for extensive follow-up information, subjects were required to have received sufficient care through the Marshfield Clinic system so that diagnosis dates for diabetes and/or breast, prostate, or colon cancer could be determined with reasonable accuracy. All subjects were required to have at least one non-diabetes diagnosis or electronic code documenting a well-visit from a Marshfield Clinic provider in at least one of the three calendar years prior to the reference date. Observation times were censored prior to any large gap in the EMR, which was defined as four or more consecutive calendar years.

Cancer diagnoses required two documented diagnoses by ICD-9 code within the EMR. The first date on which the ICD-9 code was used was considered the date of cancer diagnosis and data were merged with data from the Cancer Registry to validate diagnoses and provide additional information. Several covariates with the potential to influence cancer risk were also examined, including comorbidities and clinical risk factors, as well as used of chemotherapy and radiation during cancer treatment. Cancer treatment data were only available for subjects in the local cancer registry, which limited analyses using these data. Comorbidities of interest included myocardial infarction, coronary heart disease, peripheral vascular disease, cardiovascular disease, chronic pulmonary disease, rheumatic heart disease, and renal insufficiency/renal failure, which were summarized using a modified Charlson score (excluding cancer and diabetes). Comorbidities were established by interrogating the EMR for relevant diagnostic codes, requiring at least two documented diagnoses in the subject’s medical record. The EMR was also interrogated for body mass index (BMI), smoking history, and insurance status at reference date as well as frequency of healthcare visits before and after the reference date. For subjects with diabetes, exposure to three classes of diabetes medications including insulin, metformin, and sulfonylurea drugs was ascertained.

## Results

The process of participant selection and matching is summarized in Figure 2. Of note, application of our algorithm including laboratory parameters to the pool of potential subjects with diabetes resulted in exclusion of approximately 70% of patients. Less than 10% of potential subjects were lost when those with other diabetes-related diagnoses or abnormal glucose values > 3 years prior to diabetes diagnosis were excluded. An additional 40% of remaining potential subjects were excluded because they did not have at least two high glucose or HbA1c levels, and another 50% of potential subjects were excluded because they did not have a normal HbA1c or glucose value recorded within 3 years prior to diabetes diagnosis. After application of inclusion and exclusion criteria, there were 11,236 patients included in the final cohort with diabetes. After assigning reference dates, 54,365 participants without diabetes remained. Losses in the matching process resulted in a final matched cohort with 4.8 subjects without diabetes for each patient with diabetes, rather than the target ratio of 5:1. Despite a smaller final sample size we believe a more defined cohort is likely to be more informative and better suited for analysis than a less well-defined and refined larger cohort.

In our final validation sample, we manually abstracted evidence of diabetes diagnosis in 70 patient charts. If a diabetes diagnosis was present (N = 50), we verified the date with laboratory values for HbA1c and glucose, office notes, and medications listed. Prior records were checked to ensure that the diagnosis had not been mentioned previously but not coded. In patients in whom no diagnosis of diabetes was evident (N = 20), we verified the absence of any diabetes diagnoses on problem lists, verified that there were no high HbA1c or glucose levels, verified that no diabetes medications were listed, and that diabetes was not mentioned in the notes for a recent office visit or history and physical. In this validation sample, the observed predictive value for control subjects (NPV) was 100% (20/20). It is important to note that cases can always become controls, and this was observed in one control subject who developed diabetes in 2011—7 years after the assigned reference date in 2004—but this has no bearing on algorithm validity. The predictive value for case status (PPV) was 96% (48/50), with two subjects appearing to be incorrectly identified. However, upon arbitration, one of the two subjects was found to have a diagnosis of diabetes during the study period, increasing the positive predictive value to 98%. Overall sensitivity of the algorithm for detecting type II diabetes was 96% (95% CI 86.3–99.4%) and overall specificity was 95% (95% CI 75.1–99.2%). The date of diabetes onset determined by manual chart review was within 6 months of the study-assigned date of onset in over 70% of subjects with diabetes.

Descriptive statistics of subjects with and without diabetes are shown in Table 1. Mean observation in the EMR was similar for both groups with approximately 16 years before the reference date and 6–7 years after the reference date. The cohorts with and without diabetes were largely similar, except that BMI was higher in subjects with diabetes, and visit frequency suggested that patients with diabetes tended to have more frequent contact with the healthcare system, even before onset of diabetes. Table 1 also shows the number of patients in each cohort with a diagnosis of breast, prostate, or colon cancer.

**Table 1:** Subject descriptive characteristics by type 2 diabetes status

**Variables**	**Diabetes (N = 11,236)**	**No diabetes (N = 54,365)**
	**N (%)**	**N (%)**
Gender		
Male	5813 (51.7)	28019 (51.5)
Female	5423 (48.3)	26346 (48.5)
Mean age (years) (IQR)	62.9 (53–73)	63.2 (53–72)
Age group		
30–49 years	1940 (17.3)	9760 (18.0)
50–59 years	2681 (23.9)	12832 (23.6)
60–69 years	3110 (27.7)	14294 (26.3)
70–79 years	2409 (21.4)	10711 (19.7)
≥80 years	1096 (9.8)	6768 (12.4)
Smoking status		
Ever	7579 (67.5)	36427 (67.0)
Never	3657 (32.5)	17938 (33.0)
Diabetes diagnosis period		
1995–1999	2486 (22.1)	12123 (22.3)
2000–2004	4657 (41.4)	22581 (41.5)
2005–2009	4093 (36.4)	19661 (36.2)
MESA residency		
No	9036 (80.4)	43871 (80.7)
Yes	2200 (19.6)	10494 (19.3)
Mean BMI (kg/m^2^) (IQR)	33.4 (28–37)	28.9 (26–39)
Have insurance	8881 (79.0)	40852 (75.1)
Visit frequency during 2 years before diabetes		
0-5	2285 (20.3)	15848 (29.2)
6-10	2367 (21.1)	13093 (24.1)
11-20	3146 (28.0)	1411 (26.0)
>20	3438 (30.6)	11308 (20.8)
Visit frequency during 2 years after diabetes		
0-5	1175 (10.5)	21396 (39.4)
6-10	1523 (13.6)	10286 (18.9)
11-20	3223 (28.7)	11492 (21.1)
>20	5315 (47.3)	11191 (20.6)
Mean observation time (IQR)		
Years Before Diabetes onset	16.6 (6.1–26.1)	16.3 (5.7–26.2)
Years After Diabetes onset	7.4 (4.4–10.0)	6.1 (2.8–8.8)
Comorbidities		
Myocardial infarction	208 (1.9)	609 (1.1)
Coronary heart disease	590 (5.3)	1380 (2.5)
Peripheral vascular disease	272 (2.4)	984 (1.8)
Cardiovascular disease	351 (3.1)	1290 (2.4)
Chronic pulmonary disease	1069 (9.5)	3106 (5.7)
Rheumatic heart disease	200 (1.8)	999 (1.8)
Renal disease	207 (1.8)	713 (1.3)
Cancer		
Breast^1^	543 (10.0)	2282 (8.7)
Colon		
Men^2^	173 (3.0)	642 (2.3)
Women^1^	129 (2.4)	548 (2.1)
Prostate^2^	600 (10.3)	2832 (10.1)

IQR, interquartile range; MESA, Marshfield Epidemiologic Study Area; BMI, body mass index.

^1^Women only, N = 5,423 with diabetes and 26,346 without diabetes.

^2^Men only, N = 5,813 with diabetes and 28,019 without diabetes.

## Discussion

Several studies have examined the influence of diabetes on cancer risk and the general consensus suggests that diabetes increases cancer risk, with the notable exception of prostate cancer [10]. Diabetes is a progressive disease and physiological changes begin to occur long before clinical onset of disease [22]. During the pre-diabetes phase, patients undergo a prolonged period of increasing insulin resistance and hyperinsulinemia that ultimately results in the progressive hyperglycemia characteristic of diabetes itself. Recent evidence suggests that the hyperinsulinemia characteristic of the pre-diabetes phase is more important for promoting cancer risk than the hyperglycemia present after clinical onset [13,14]. Despite this evidence, examining cancer risk in the pre-diabetes phase is difficult and the temporal relationship between the two diseases has remained largely unexplored. We developed an electronic algorithm that calls upon administrative, laboratory, and clinical data to accurately identify patients with type 2 diabetes and to determine the date of clinical onset for over 10,000 patients. A cohort without diabetes was also generated and includes over 50,000 patients with assigned reference dates. Together, the cohort of approximately 65,000 patients with over 16 years of follow-up after diabetes onset and 6–7 years of observation before provides a resource for the temporal examination of the relationship between diabetes and cancer risk.

Previous studies regarding the relationship between diabetes and cancer relied on algorithms to identify diabetes that were heavily reliant on self-report and/or administrative data, limited in their ability to distinguish between type 1 and 2 diabetes, and unable to accurately determine date of diabetes onset [23,24]. Several algorithms have recently been developed to make use of EMR data to accurately identify patients with type 2 diabetes primarily for surveillance purposes. Kuydakov et al. [25] developed an algorithm for identifying newly diagnosed cases of type 2 diabetes with the main criterion being a minimum 30-day window between the first documented visit in the EMR and entry of type 2 diabetes in the problem list. However, this method fails to account for the potential for subjects to have diabetes for long periods of time before a diagnosis is made. Other algorithms have used additional EMR data to capture patients with diabetes even before diagnosis using various combinations of laboratory results and prescription information in addition to diagnostic and billing codes [19,26-29]. While these algorithms perform well for their intended purposes, none serve to accurately identify the date of diabetes onset and are thus unsuitable for examination of the temporal relationship between diabetes and cancer, which requires clear delineation of the time periods before and after diabetes onset. The method described here focuses on determining date of clinical diabetes onset as accurately as possible using a combination of laboratory results and diagnostic codes in addition to time limits to ensure capture of onset within a three-year time window. Table 2 offers a side-by-side comparison of these algorithms. The element of time is of particular importance to the algorithm designed for the present study. For a patient to be classified as having diabetes, abnormal laboratory values (HbA1c or glucose) were required to occur within three years of a normal laboratory values, suggesting that clinical onset of diabetes occurred in the interim. Date of onset was defined as the earlier of the first diabetes diagnosis code or second high laboratory result, clearly delineating the time periods before and after diabetes onset for temporal examination. Validation of a similar Marshfield Clinic EMR-based algorithm for identification of patients with and without type 2 diabetes showed a 99% predictive value for type 2 cases and 98% for type 2 controls [30]. Results were similar using the algorithm described here with a 98% predictive value for type 2 cases and 100% for type 2 controls.

**Table 2:** Comparison of algorithms using electronic medical record data for identification of patients with type 2 diabetes

	**EMR elements**				
**Reference**	**Billing/Diagnostic codes**	**Laboratory results**^**1**^	**Medications**	**Timeframe**	**Diabetes onset**
[23]	≥ 1 short-stay hospital, skilled nursing facility, or home health agency claim or ≥ 2 physician/supplier claims with diabetes diagnosis	--	--	1 – 2 year identification period	--
[24]	1 hospital discharge abstract or 2 physician services claims showing diabetes	--	--	2 year period	--
[19]	250.X0, 250.X2, 357.2, 362.0X, 583.81	≥ 1 high HbA1c or random glucose or ≥ 2 random glucose tests	Metformin, sulfonylurea, or insulin	--	--
[25]	250.X0, 250.X2, or 362.XX (no insulin)	≥ 1 high HbA1c or ≥ 2 high fasting or random glucose tests	--	Diagnosis ≥ 30 days after first office visit	First appearance in problem list
[26]	On problem list (coded or free text) ≥ 2 times in 2 years	≥ 2 high fasting glucose tests in 1 year or any high HbA1c^2^	Hypoglycemic medications	--	--
[27]	250.X	High HbA1c, fasting, or random glucose^3^	Number unique anti-diabetes medications	--	--
[28]	≥ 2 250.X0 or 250.X2	High HbA1c or fasting glucose test	Insulin or oral hypoglycemic agents except metformin	Surveillance in real time	--
[29]	No type 1 code, ≥ 2 type 2 codes	Abnormal glucose or HbA1c	Type 2 diabetes medications	--	--
Current Study	≥ 1 250.X0 or 250.X2, ≥ 1 year before any type 1 code	≥ 2 high HbA1c or glucose test and ≥ 1 normal HbA1c or glucose test	Excluded for diabetes medication > 30 days before diagnosis	Normal and abnormal labs within 3 years	Earliest of first diagnosis or second high lab

HbA1c, glycated hemoglobin.

^1^Laboratory values coincide with American Diabetes Association guidelines (HbA1c ≥ 6.5%, fasting blood glucose ≥ 126 mg/dl, random blood glucose ≥ 200 mg/dl) [20] unless otherwise specified.

^2^Fasting blood glucose > 7 mmol/L, HbA1c ≥ 7%.

^3^HbA1c > 6%, fasting or random glucose > 110 mg/dl.

Despite meticulous selection of patients with and without diabetes for study inclusion, our cohort is nevertheless subject to biases inherent to retrospective and observational studies as well as certain time-related biases common in observational studies. In addition, the data available in an EMR are only as good as the data input during routine patient care. As such, for healthcare systems in which several healthcare choices are available nearby, laboratory values and diagnoses captured outside of the healthcare system may not be available. Marshfield Clinic serves a relatively rural, agriculture-based population with little turnover and little choice of healthcare provider. As such, our EMR serves as a robust source of data, but we recognize that certain data points may be missing. Ascertainment bias is inherent to retrospective studies. In the current cohort, ascertainment bias may result from the fact that patients with diabetes have more frequent contact with the healthcare system. Additionally, HbA1c screening was not recommended by the ADA until 2010, after the study period, and in the 3 years prior it is estimated that only 10–20% of adults without diabetes underwent HbA1c testing [31], which may introduce an additional source of ascertainment bias. As with other laboratory tests, methods for measuring HbA1c have also changed over time. Use of reference period as a matching criterion in cohort development is likely to minimize the effects of any such change, however. Similarly, selection bias may result from the unintentional selection for differing characteristics among patients who, for example, receive a particular diabetes treatment. Additionally, labs drawn to assess HbA1c and glucose levels may be more likely to be performed in patients with a higher risk for cancer, of concern in both groups. The effects of selection bias are minimized to some extent by the cohort design, which uses an extensive matching process to account for age, gender, residence, smoking history, and reference period. In future work using the cohort described here, the potential for additional confounding as a result of selection bias will be minimized via proportional hazards regression modeling with adjustment for relevant covariates. Time-related biases, including immortal time bias, time-window bias, and time-lag bias, will be of particular concern when considering the effect of exposure to diabetes medications on cancer risk [32]. While elimination of such biases may not be realistic, efforts to minimize their effects include use of time-varying analyses, assessment of follow-up time, and examination of both exposure and duration in analyses of diabetes medications. Importantly, reference period was used as a matching criterion in cohort development and follow-up time before and after the reference date or date of diabetes onset was similar.

## Conclusion

Murdoch and Detsky recently reported on the inevitable application of the massive amount of data captured by the EMR to health care, emphasizing the potential value of using information generated in the course of routine care to answer important questions and to improve the quality of care [33]. Here we demonstrate an example using data abstracted electronically from the EMR to develop patient cohorts for the careful examination of the temporal relationship between diabetes and cancer. To date, the cohort described here has been used to examine the temporal relationship between diabetes and breast cancer in women [34], prostate cancer in men [35], and colon cancer [36], as well as the effects of glycemic control and medication exposure on cancer risk [37]. In the future, we plan to use this cohort to examine tumor severity and survival as well as the effects of additional disease conditions and comorbidities, such as sleep apnea, on cancer risk in patients with diabetes.

## Abbreviations

EMR: Electronic medical record; PMRP: Personalized medicine research project; MESA: Marshfield epidemiologic study area; ICD-9: International classification of disease, version 9; HbA1c: Glycated hemoglobin; BMI: Body mass index.

## Competing interests

The authors declare that they have no competing interests.

## Authors’ contributions

AAO, RVS, RLB, and JME contributed to study conception and design. AAO and RLB assessed data and performed statistical analyses. AAO, GMW, and SAD supervised and coordinated the project. RVS drafted the manuscript, which was edited and revised by AAO, JME, and RLB. All authors read and approved the final manuscript.

## Pre-publication history

The pre-publication history for this paper can be accessed here:

http://www.biomedcentral.com/1472-6947/14/38/prepub
